# Relationship between Personality Traits and Nicotine Dependence in Male and Female Smokers of African-American and European-American Samples

**DOI:** 10.3389/fpsyt.2017.00122

**Published:** 2017-07-18

**Authors:** Jung-Seok Choi, Thomas J. Payne, Jennie Z. Ma, Ming D. Li

**Affiliations:** ^1^Department of Psychiatry and Neurobehavioral Sciences, University of Virginia, Charlottesville, VA, United States; ^2^Department of Psychiatry, SMG-SNU Boramae Medical Center, Seoul National University College of Medicine, Seoul, South Korea; ^3^Department of Psychiatry and Behavioral Science, Seoul National University College of Medicine, Seoul, South Korea; ^4^Department of Otolaryngology and Communicative Sciences, University of Mississippi Medical Center, Jackson, MS, United States; ^5^Department of Public Health Sciences, University of Virginia, Charlottesville, VA, United States

**Keywords:** nicotine dependence, personality, neuroticism, conscientiousness, tobacco smoking

## Abstract

**Aims:**

Personality characteristics are linked to nicotine dependence (ND). It remains unclear whether these factors differ across African-American (AA) and European-American (EA) male and female smokers. This study was aimed to determine the relationship between personality traits and smoking status, as well as the degree of ND, in AA and EA male and female samples.

**Methods:**

A total of 5,040 participants (AA: *N* = 3,737, female = 54.31%; EA: *N* = 1,313, female = 64.51%) were included in this study, with 2,474 smokers and 2,566 non-smokers. The measures used in this study included five dimensions of personality by the NEO-personality inventory-revised (neuroticism, extraversion, agreeableness, openness to experience, and conscientiousness), Fagerström test for ND, and drive subscale of the ND Syndrome Scale (NDSS).

**Results:**

In the AA sample, neuroticism was significantly associated with a higher risk of smoking [odds ratio (OR) = 1.057; 95% confidence interval (CI) 1.032, 1.083; *p* < 0.0001], and conscientiousness was significantly associated with decreased risk of smoking (OR = 0.936; 95% CI 0.912, 0.961; *p* < 0.0001). In the EA sample, higher neuroticism was associated with increased risk of being a current smoker (OR = 1.058; 95% CI 1.013, 1.104; *p* = 0.0105). Furthermore, we found that a lower level of neuroticism and higher level of conscientiousness were associated with the severity of ND in both the AA and EA samples and a broader range of personality factors were involved in predicting the severity of ND in the AA samples. However, no differential association was detected between male and female smokers of both AA and EA samples.

**Conclusion:**

There exist differential relationships between personality traits and the severity of ND in the AA and EA samples.

## Introduction

Even though 67% of regular smokers have considered quitting and 52% have attempted to do so during the past year (1, 2), most efforts to remain abstinent for at least 6 months are unsuccessful (3, 4). Various biological, psychological, and social factors have been implicated in difficulties achieving and maintaining abstinence (5). Elucidating phenotypes linking such factors with smoking behaviors may enhance our capacity to develop tailored treatment strategies for treating nicotine dependence (ND).

Personality traits have been the subject of sustained attention in mediating the development and presentation of ND (6). Strong evidence is available attesting to this relationship. For example, in a meta-analysis of cross-sectional studies, smokers had higher neuroticism and extraversion scores than non-smokers (7). Another meta-analysis revealed that smokers scored higher on neuroticism and extraversion and lower on conscientiousness than non-smokers, and increased likelihood of relapse to smoking was associated with higher neuroticism (6). In addition, daily smokers, as compared with former and never smokers, tend to score higher in neuroticism (8, 9). Increased neuroticism is associated with a higher rate of smoking as a means for managing negative moods, as well as poorer cessation outcomes (10). Smoking initiation in adulthood is predicted by a lower level of conscientiousness in childhood (11); furthermore, a lower level of agreeableness is often associated with higher risk of smoking (12). Some studies have noted that cigarette smokers score higher on extraversion (7, 12, 13) and openness to experience (14) than do non-smokers. The health behavior model of personality is one of the leading theories that addresses the role of personality in determining an individual’s health (15, 16). According to this model, certain personality traits (particularly conscientiousness and neuroticism) are associated with either health promoting or health debilitating behaviors (e.g., smoking, drinking, and/or drug use), thereby determining health outcomes (16–18). In essence, health behaviors mediate the relationship between personality and both morbidity and mortality (19). Thus, this relatively consistent body of findings indicates the presence of relationship between higher neuroticism and lower conscientiousness and smoking status.

Recent literature has revealed ethnic and sex differences in smoking behavior. Although the prevalence of current smoking among adults is similar across European-Americans (EA; 16.6%) and African-Americans (AA; 16.7%) (20), men are more likely to be current tobacco smokers than women in the USA, i.e., 16.7% of men and 13.6% of women (20), and there also exist ethnic differences in time to first cigarette (TTFC), one of the best indicators of ND (21). A lower TTFC score that indicates earlier cigarette smoking after awakening is associated with higher levels of relapse, nicotine withdrawal symptoms, nicotine intake, tobacco carcinogen exposure, and cancer risk in adolescent and adult smokers (21–26). AA smokers are more likely to smoke within 5 min of awakening than EAs (21). AA smokers report greater abstinence-induced declines in positive affect states, compared with EA smokers (27). Moreover, AA (vs. EA) and female (vs. male) may be at greater risk for relapse following a cessation attempt (28, 29).

Comparatively little research exists on how the personality factors referenced above differ across male and female smokers of AA or EA population and whether these differences influence ethnic or sex differences in smoking-related behavior. Most previous research has examined the relationship between personality features and smoking status, such as smoking initiation, cessation, or relapse (30, 31). Furthermore, the association between personality and smoking status is stronger among females than males (32). However, little evidence exists regarding whether personality features differentially influence the severity of ND across ethnicity and sex. Such evidence may provide insight into the development of tailored prevention and intervention programs. Identifying higher risk individuals based on particular combinations of personality traits, sex, and ethnicity would have implications for a more severe level of ND, requiring more aggressive treatment, as well as adjusting counseling options to maximize the impact of treatment on those individuals.

While the Fagerström test for nicotine dependence (FTND) is known to emphasize the role of physiological factors (33, 34), the drive subscale of the ND Syndrome Scale (NDSS) provides additional information regarding the severity of craving and withdrawal symptoms experienced when attempting to quit, as well as the likelihood of relapse (35). Thus, simultaneous consideration of these complementary instruments may improve our understanding and predictions related to tobacco use and cessation.

In the present cross-sectional study, we investigated the relationship between personality traits and smoking status, as well as the severity of ND across male and female smokers of AA or EA ethnicity. Furthermore, we examined the relationship between personality traits and the degree of craving and withdrawal symptoms as a function of TTFC. We hypothesized that higher neuroticism and lower conscientiousness would be related to a higher probability of smoking, as well as increased severity of ND. Furthermore, we hypothesized elevated levels of targeted personality traits would influence the severity of ND differentially for AA vs. EA and female vs. male smokers.

## Materials and Methods

### Participants

Detailed information about study participants has been provided in a previous report (36). Briefly, data were extracted from the records of individuals enrolled in a large genetics study of ND conducted by our team during 1999–2012. Selection criteria of subjects included a minimum age of 18, EA or AA ethnicity, no history of substance dependence other than nicotine or alcohol in the past 12 months, and no history of diagnosis of any serious mental disorder (e.g., psychotic disorder, severe cognitive problems). In addition, proband smokers were required to report daily cigarette use ≥20 for at least the past 5 years and produce an expired carbon monoxide (CO) concentration ≥8 ppm. Non-smoker selection criteria included 1–99 cigarettes lifetime, no history of regular use of any other tobacco product, no use of any tobacco product or tobacco treatment medications in the past year, and an expired CO concentration of <8 ppm. Those individuals who agreed to participate were screened, provided informed consent, and completed questionnaires either on paper or on a touch-screen laptop running a custom Microsoft Access application requiring approximately 45 min to complete.

A total of 5,040 subjects who completed smoking and personality questionnaires were included in this study. The study received Institutional Review Board approval from all participating institutions. Informed consent was obtained from all participants in accordance with approved procedures.

### Measures

Questionnaires administered addressed a broad range of topics related to the goals of the main project. For this study, the following measures were selected: (a) demographic characteristics, including age, sex, ethnicity, education, and income; (b) FTND (37), a commonly used instrument to measure severity of ND (total score range 0–10, α = 0.75) (36); (c) TTFC (an item from the FTND, categorized as 0: >60 min; 1: 31–60 min; 2: 6–30 min; 3: ≤5 min); (d) drive subscale of the NDSS (35) to assess craving and withdrawal symptoms; and (e) the NEO-personality inventory-revised (NEO-PI-R) (38). The NEO-PI-R assesses personality characteristics consistent with the five-factor model of personality: neuroticism (tendency to experience negative emotions), extraversion (sociability and assertiveness), openness to experience (creativity, adventurousness, and receptivity to new ideas), agreeableness (degree to which behavior is compliant and cooperative), and conscientiousness (self-discipline and organization) (38). These five traits are heritable, stable over time, and have been shown to be generalizable across diverse social contexts (39, 40). For example, a general heritable component is associated with all five personality traits (estimates ranging from path coefficients = 0.30–0.55) (41) and the heritability for neuroticism and openness to experience is estimated to be 15 and 21%, respectively (39). Reliability Cronbach’s alpha coefficients in this study are as follows: neuroticism (α = 0.70), extraversion (α = 0.70), openness to experience (α = 0.64), agreeableness (α = 0.80), and conscientiousness (α = 0.70).

### Statistical Analysis

Comparisons of demographic and clinical characteristics were conducted using analysis of variance for continuous variables and chi-square tests of independence for categorical variables. Associations between personality traits and smoking status were analyzed using multiple logistic regressions, adjusting for demographic characteristics, including age, sex, ethnicity, education, and family income. In the regression model, five factors of personality were all included as independent variables. Associations between personality traits and smoking-related measures (FTND, TTFC, and NDSS drive) for smokers only were analyzed using a multivariate linear regression (42), which handles multiple outcomes and incorporates correlation between outcomes from the same subject. In this analysis, unstructured variance–covariance structure was considered for correlation between outcomes. Consistent with the logistic regression, demographic characteristics and five factors of personality were used as covariates in the model. Because our main interest concerned the associations between personality traits and smoking-related characteristics by ethnicity and sex, interaction effects among different smoking-related measures, ethnicity, sex, and personality traits were examined in the model. If the highest interaction terms among types of smoking-related measures, ethnicity, sex, and personality traits were significant, the relationships between each personality trait and smoking-related measures were examined using contrast tests controlling ethnicity and sex. On the other hand, if the highest interaction terms were not significant, the final model was selected based on the likelihood ratio test (LRT) and Bayesian information criterion (BIC) (43). From the model results, differential associations between personality traits and smoking-related measures were estimated using contrast tests. A *p*-value of <0.05 was considered significant in the multivariate regression model. In case of contrast tests, *p* < 0.01 was considered significant after adjusting for multiple comparisons of five personality traits to minimize type I errors. All statistical analyses were performed with IBM SPSS Statistics version 20 (IBM Inc., Chicago, IL, USA) and R package nlme (44) of R version 3.3.2 (http://www.r-project.org).

## Results

### Sample Characteristics

A total of 5,040 participants were included in this study, comprised of 2,474 smokers (49%) and 2,566 non-smokers (51%). The sample was 57% women and 74% of AA origin. As shown in Table 1 AA smokers were slightly older than non-smokers (43.63 ± 12.59 vs. 42.22 ± 14.26 years; *p* = 0.0131), whereas EA non-smokers were older than smokers (46.98 ± 14.76 vs. 42.59 ± 12.45 years; *p* < 0.0001). Non-smokers of both ethnicities were more likely to have achieved education beyond high school than were smokers (AAs: 35.63 vs. 29.07%; *p* < 0.0001; EAs: 36.75 vs. 27.30%; *p* = 0.0003). EA non-smokers were more likely to earn $30,000 or more per year than EA smokers (63.10 vs. 44.92%; *p* < 0.0001); there was no difference for AA participants. EA smokers scored higher than AA smokers on the FTND (8.73 ± 1.14 vs. 8.61 ± 1.06; *p* < 0.0001) and reported greater CPD than AA smokers (27.97 ± 6.72 vs. 25.76 ± 5.73; *p* < 0.0001); although the absolute magnitude of these differences was relatively small. No significant differences across ethnicity were observed for TTFC or NDSS Drive.

**Table 1:** Demographic and clinical characteristics of AA and EA samples used in the study.

	AA			EA		
	Smokers	Non-smokers	*p*	Smokers	Non-smokers	*p*
				
	(*n* = 1,844) mean ± SD	(*n* = 1,883) mean ± SD		(*n* = 630) mean ± SD	(*n* = 683) mean ± SD	
Age	43.63 ± 12.59	42.22 ± 14.26	0.0131	42.59 ± 12.45	46.98 ± 14.76	<0.0001
Sex (M/F, *n*)	915/929	788/1095	<0.0001	234/396	232/451	0.2297
≥HS education (%)	29.07	35.63	<0.0001	27.30	36.75	0.0003
Income ≥$30K (%)	41.65	42.27	0.6994	44.92	63.10	<0.0001

**Smoking-related variables**						

CPD	25.76 ± 5.73			27.97 ± 6.72		<0.0001
FTND	8.61 ± 1.06			8.73 ± 1.14		<0.0001
TTFC	2.84 ± 0.42			2.85 ± 0.44		0.8214
NDSS, drive subscale	−0.92 ± 0.42			−0.92 ± 00.39		0.9113

**Personality-related variables**						

Neuroticism	33.82 ± 3.06	33.11 ± 3.03	<0.0001	33.95 ± 3.20	33.23 ± 2.67	<0.0001
Extraversion	39.34 ± 2.82	39.96 ± 2.85	<0.0001	39.64 ± 2.80	39.44 ± 2.53	0.1667
Openness to experience	38.47 ± 2.36	38.85 ± 2.30	<0.0001	38.74 ± 2.29	38.42 ± 2.24	<0.0001
Agreeableness	37.32 ± 3.23	37.11 ± 3.05	0.0851	37.42 ± 3.26	36.82 ± 2.81	0.0004
Conscientiousness	42.78 ± 3.63	43.74 ± 3.10	<0.0001	43.06 ± 3.22	43.15 ± 3.04	0.5942

*AA, African-Americans; EA, European-Americans; HS, high school; FTND, Fagerström test for nicotine dependence; CPD, number of cigarettes per day; TTFC, time to first cigarette; 0 (>60 min), 1 (31–60 min), 2 (6–30 min), 3 (≤5 min); NDSS, ND Syndrome Scale; *p* < 0.05*.

African-Americans smokers revealed higher neuroticism (33.82 ± 3.06 vs. 33.11 ± 3.03; *p* < 0.0001), lower extraversion (39.34 ± 2.82 vs. 39.96 ± 2.85; *p* < 0.0001), lower openness to experience (38.47 ± 2.36 vs. 38.85 ± 2.30; *p* < 0.0001), and lower conscientiousness (42.78 ± 3.63 vs. 43.74 ± 3.10; *p* < 0.0001) than AA non-smokers, whereas EA smokers showed higher neuroticism (33.95 ± 3.20 vs. 33.23 ± 2.67; *p* < 0.0001), higher openness to experience (38.74 ± 2.29 vs. 38.42 ± 2.24; *p* = 0.0101), and higher agreeableness (37.42 ± 3.26 vs. 36.82 ± 2.81; *p* = 0.0004) than EA non-smokers (Table 1). The absolute magnitude of the differences was small. There were no significant differences in personality traits between the AA and EA smokers.

When we examined AA and EA smokers with respect to sex (Table 2), both AA and EA male smokers were older than female smokers (AAs: 44.65 ± 12.41 vs. 42.63 ± 12.69 years; *p* = 0.0006; EAs: 45.38 ± 12.38 vs. 40.93 ± 12.20 years; *p* < 0.0001). AA female smokers were more likely to have achieved education beyond high school relative to AA male smokers (32.08 vs. 26.01%; *p* = 0.0041). Male smokers of both ethnicities were more likely to earn $30,000 or more per year than female smokers (AAs: 49.84 vs. 33.58%; *p* < 0.0001; EAs: 53.42 vs. 39.90%; *p* = 0.0010). In both AA and EA samples, male smokers produced higher scores on the FTND (AAs: 8.75 ± 0.94 vs. 8.47 ± 1.15; *p* < 0.0001; EAs: 8.94 ± 0.90 vs. 8.61 ± 1.24; *p* = 0.0004) and indicated higher CPD (AAs: 26.67 ± 5.91 vs. 24.87 ± 5.41; *p* < 0.0001; EAs: 29.36 ± 7.00 vs. 27.14 ± 6.42; *p* < 0.0001) than female smokers. On the other hand, there were no significant sex differences in personality traits for either AA or EA sample (Table 2).

**Table 2:** Demographic and clinical characteristics of AA and EA smokers according to gender in the study.

	AA smokers			EA smokers		
	Male (*n* = 915) mean ± SD	Female (*n* = 929) mean ± SD	*p*	Male (*n* = 234) mean ± SD	Female (*n* = 396) mean ± SD	*p*
Age	44.65 ± 12.41	42.63 ± 12.69	0.0006	45.38 ± 12.38	40.93 ± 12.20	<0.0001
≥HS education (%)	26.01	32.08	0.0041	28.63	26.52	0.5644
Income ≥$30K (%)	49.84	33.58	<0.0001	53.42	39.90	0.0010

**Smoking-related variables**						

CPD	26.67 ± 5.91	24.87 ± 5.41	<0.0001	29.36 ± 7.00	27.14 ± 6.42	<0.0001
FTND	8.75 ± 0.94	8.47 ± 1.15	<0.0001	8.94 ± 0.90	8.61 ± 1.24	0.0004
TTFC	2.88 ± 0.37	2.81 ± 0.46	0.0002	2.89 ± 0.33	2.82 ± 0.49	0.0605
NDSS, drive subscale	−0.91 ± 0.40	−0.93 ± 0.43	0.2502	−0.92 ± 00.34	−0.92 ± 00.42	0.9214

**Personality-related variables**						

Neuroticism	33.74 ± 3.02	33.90 ± 3.10	0.2553	33.79 ± 2.95	34.04 ± 3.34	0.3579
Extraversion	39.36 ± 2.66	39.32 ± 2.98	0.7370	39.69 ± 2.38	39.62 ± 3.03	0.7563
Openness to experience	38.56 ± 2.21	38.38 ± 2.49	0.1198	38.68 ± 2.22	38.79 ± 2.34	0.5608
Agreeableness	37.39 ± 3.16	37.25 ± 3.31	0.3586	37.23 ± 3.42	37.53 ± 3.17	0.2633
Conscientiousness	42.86 ± 3.46	42.70 ± 3.79	0.3431	43.35 ± 3.07	42.89 ± 3.29	0.0864

*AA, African-Americans; EA, European-Americans; HS, high school; FTND, Fagerström test for nicotine dependence; CPD, number of cigarettes per day; TTFC, time to first cigarette; 0 (>60 min), 1 (31–60 min), 2 (6–30 min), 3 (≤5 min); NDSS, ND Syndrome Scale*.

### Personality Factors and Smoking-Related Variables

As shown in Table 3, higher neuroticism and agreeableness were associated with an increased likelihood of being a current smoker among AAs (OR = 1.057; 95% confidence interval (CI) 1.032, 1.083; *p* < 0.0001; OR = 1.035; 95% CI 1.010, 1.060; *p* = 0.0057, respectively), whereas higher conscientiousness was associated with lower likelihood of being a current smoker (OR = 0.936; 95% CI 0.912, 0.961; *p* < 0.0001). In the EA sample, higher neuroticism was associated with increased likelihood of being a current smoker (OR = 1.058; 95% CI 1.013, 1.104; *p* = 0.0105), although this relationship was non-significant after correction for multiple comparisons. Furthermore, when using a multivariate linear regression to investigate associations between personality traits and severity of ND, the overall fourth interaction terms using contrast tests were not significant (*F* = 1.263, *p* = 0.2456). According to the LRT and BIC, the reduced model was selected, as presented in Table 4. In the final model, third-order interaction effects were significant (*F* = 2.218, *p* = 0.0166). This indicates that at least one smoking-related measure showed the differential relation with personality trait by ethnicity. No differential relation across sex was identified. The contrast test results on the associations between personality traits and severity of ND are presented in Table 3.

**Table 3:** Significant predictive personality factors for smoking-related variables in each AA and EA smokers.

Sample	Personality	Smoker		Nicotine dependence					
				FTND		TTFC		NDSS drive	
		Odds ratio (95% confidence interval)	*p*	β	*p*	β	*p*	β	*p*
AA	Neuroticism	1.057 (1.032−1.083)	<0.0001	−0.049	<0.0001	−0.023	<0.0001	−0.012	0.0002
	Extraversion	0.964 (0.933−0.996)	0.0269	−0.011	0.3564	−0.001	0.7906	0.001	0.8179
	Openness to experience	0.988 (0.951−1.027)	0.5443	0.028	0.0401	0.015	0.0047	0.009	0.0881
	Agreeableness	1.035 (1.010−1.060)	0.0057	−0.045	<0.0001	−0.008	0.0114	−0.007	0.0352
	Conscientiousness	0.936 (0.912−0.961)	<0.0001	0.082	<0.0001	0.030	<0.0001	0.024	<0.0001
EA	Neuroticism	1.058 (1.013−1.104)	0.0105	−0.052	0.0002	−0.016	0.0029	−0.011	0.0474
	Extraversion	1.027 (0.972−1.084)	0.3499	0.010	0.5724	0.006	0.4417	0.011	0.1277
	Openness to experience	1.072 (1.005−1.144)	0.0361	−0.058	0.0186	−0.010	0.2684	−0.009	0.2767
	Agreeableness	1.029 (0.987−1.073)	0.1818	−0.004	0.7565	−0.006	0.3083	−0.002	0.7089
	Conscientiousness	0.977 (0.933−1.023)	0.3180	0.085	<0.0001	0.032	<0.0001	0.017	0.0045

*AA, African-Americans; EA, European-Americans; FTND, Fagerström test for nicotine dependence; TTFC, time to first cigarette; NDSS, ND Syndrome Scale; p < 0.01, adjusted for multiple correction*.

**Table 4:** Interactions among personality, ethnicity, sex, and smoking-related measurements in smokers.

Variables	(NumDF, DenDF)	*F*-statistic	*p*-Value
Type of smoking-related measures	(2, 2,458)	75.779	<0.0001
Sex	(1, 2,458)	40.137	<0.0001
Personality	(5, 2,458)	13.917	<0.0001
Ethnicity	(1, 2,458)	1.144	0.2849
Type of smoking-related measures × sex	(2, 2,458)	23.453	<0.0001
Type of smoking-related measures × ethnicity	(2, 2,458)	0.624	0.5361
Type of smoking-related measures × personality	(10, 2,458)	5.587	<0.0001
Ethnicity × personality	(5, 2,458)	3.324	0.0054
Type of smoking-related measures × ethnicity × personality	(10, 2,458)	2.178	0.0166

Among the AA smokers, four types of personality traits (i.e., neuroticism, openness to experience, agreeableness, and conscientiousness) were associated with severity of ND. Lower neuroticism was associated with higher FTND score (β = −0.049; *p* < 0.0001), TTFC (β = −0.023; *p* < 0.0001), and NDSS Drive (β = −0.012; *p* = 0.0002). Higher conscientiousness was associated with a higher FTND score (β = 0.082; *p* < 0.0001), TTFC (β = 0.030; *p* < 0.0001), and drive subscale score (β = 0.024; *p* < 0.0001). Among the EA smokers, lower neuroticism was associated with a higher FTND score (β = −0.052; *p* = 0.0002) and TTFC (β = −0.016; *p* = 0.0029), whereas higher conscientiousness was associated with a higher FTND score (β = 0.085; *p* < 0.0001), TTFC (β = 0.032; *p* < 0.0001), and NDSS Drive (β = 0.017; *p* = 0.0045).

Next, we analyzed the relationship between personality traits and NDSS drive as a function of TTFC. Among the AAs who smoked within 5 min after waking (≤5 min of TTFC: category 3), conscientiousness was significantly associated with NDSS drive (β = 0.163; *p* < 0.0001); no significant associations emerged for other TTFC categories, such as 6–30, 31–60, or >60 min. For EAs, conscientiousness was associated with the NDSS drive (β = 0.118; *p* = 0.0177) in the TTFC (≤5 min category); this effect was no longer significant after multiple correction. There were no significant associations between personality factors and NDSS drive for EA smokers in the TTFC 6–30, 31–60, or >60 min categories.

## Discussion

The results emerging from this study indicate that neuroticism and conscientiousness are associated with the likelihood of being a current smoker, as well as level of ND. Furthermore, personality traits have a greater influence on smoking status and severity of ND in AAs relative to EAs. These relationships were particularly pronounced among smokers with reporting TTFC of ≤5 min.

Both AA and EA smokers reported a higher level of neuroticism relative to non-smokers and AA smokers scored lower on conscientiousness, extraversion, and openness to experience relative to non-smokers; however, EA smokers showed higher openness to experience and agreeableness relative to non-smokers. Consistent with a previous report (45), we found that higher neuroticism and lower conscientiousness were associated with higher likelihood of being a current smoker in the AA sample. Individuals who demonstrate higher levels of neuroticism have been shown to employ smoking as a coping strategy for emotional regulation (46); those scoring lower on conscientiousness more likely engage in activities that pose health risks (47). Therefore, higher neuroticism and lower conscientiousness may increase one’s risk of progression to daily smoking *via* various reinforcement mechanisms.

Interestingly, both of these scales revealed an inverse association with ND as measured by FTND and TTFC, and likelihood of increased craving/withdrawal symptoms as indicated by the NDSS drive subscale. This represents a mechanism that may help explain the risk for heavier smoking. This differential response at higher level of ND is consistent with our recent report indicating a higher, clinically meaningful level of depressive symptoms for smokers relative to non-smokers, with daily cigarette smoking rate inversely associated with depressive symptom severity among heavy smokers (36). These findings extend our previous results, suggesting that heavy tobacco users display characteristics and symptoms indicative of increased distress but these symptoms may be more effectively ameliorated by higher levels of tobacco intake.

We found differential patterns of association between conscientiousness and NDSS drive for EA and AA heavy smokers reporting TTFC ≤5 min. This relationship for EA smokers was non-significant after correction for multiple comparisons. Thus, among more highly nicotine-dependent individuals, increased conscientiousness is associated with abstinence symptom severity as well as relapse risk, particularly for AA smokers. This relationship may help identify those individuals who will have the greatest difficulty achieving and maintaining abstinence. Personality traits are heritable and influenced by genetic components (39, 48). A recent study reported that higher neuroticism and lower conscientiousness were associated with genetic polymorphism of the dopaminergic system (48). Furthermore, personality traits could influence the sensitivity to the reinforcing effects of psychostimulants, including nicotine (46). The mechanism for this differential relationship among heavy smokers is unclear at the present time but may include differential physiological responses as a function of such genetic components or other processes. Thus, it is necessary to clarify the relationship between genetic factors, personality traits, and smoking-related behaviors in light vs. heavy smokers.

Among AA smokers, neuroticism, conscientiousness, openness to experience, and agreeableness were associated with the FTND or NDSS drive, whereas only neuroticism and conscientiousness demonstrated such relationships for EA smokers. Various combinations of personality factors may interact each other, leading to certain individuals becoming prone to engaging in health-damaging substance use behaviors (19). The broader range of relationships with personality factors for AA smoker suggests greater difficulty in quitting and maintaining abstinence, potentially as a function of additional mechanisms/pathways for influence. Some pieces of evidence suggest that relative to EA, AA smokers display lower cessation rates (i.e., a lower proportion of former smokers among ever-smokers) (47). Therefore, it is possible that more elaborate associations between personality traits and ND among AA smokers may render tobacco use a more tenacious addiction.

Previous studies have reported that the association between personality and smoking was either stronger among females than males (32) or showed no difference (19, 49). Hampson et al. (32) reported that childhood personality traits were significantly associated with smoking status in adults, particularly for girls. Jerram and Coleman (50) found that agreeableness and openness to experience were the most important predictors of various health measures for women. Contrary to our hypotheses, however, there were no significant differences in personality traits between male and female of both AA and EA samples as well as no associations between personality and severity of ND according to sex in this study. Sex differences in personality can be affected by different cultures, which can lead to inconsistent findings (51). Furthermore, this study was cross-sectional in design; longitudinal assessments will be necessary to evaluate the associations between personality traits and later smoking-related behaviors.

This study has limitations. Because our samples were composed of AA and EA smokers residing in Southeastern United States, generalizability to other populations or locations should be considered tentative until additional evidence is available. In addition, most smokers in this study were highly nicotine dependent, so whether these relations would apply to lighter smokers is unknown. Sample sizes in some of our subgroups were smaller, which may have had some impact on statistical power. The study design is cross-sectional and it is difficult to clarify the causal relationship between personality traits, ND, and smoking status. Finally, the overall magnitude of the associations with personality dimensions was modest; thus, these effects must be interpreted within the context of the wide range of other important factors determining tobacco use, such as physiological, social, environmental, tobacco products, and public policy considerations.

In summary, the present results indicate that personality traits are associated with smoking status and degree of ND and these relations are moderated by ethnicity. Personality factors appear to have greater influence in AAs relative to EAs. In particular, for AA smokers, neuroticism, conscientiousness, openness to experience, and agreeableness were associated with degree of ND, but only neuroticism and conscientiousness for EA smokers. This work has implications for enhancing our understanding of important clinical issues for heavy smokers with regard to the role of personality. Based on considerations of interaction among personality traits and ethnicity, individualized treatment approach should be adopted for smoking cessation through modifying health behaviors. Further research is necessary to clarify the relationship among genetic factors, personality traits, and smoking behaviors.

## Ethics Statement

This study was carried out in accordance with the recommendations of NIH guidelines with written informed consent from all subjects. All subjects gave written informed consent in accordance with the Declaration of Helsinki. The protocol was approved by the Institutional Reviewing Board of University of Virginia and University of Mississippi Medical Center.

## Author Contributions

J-SC performed data analysis and paper writing; TP participated subject recruitment, data collection, and paper writing; JM involved in data collection, data management, data analysis, and paper writing; ML designed study and involved in subject recruitment, data collection, and paper writing.

## Conflict of Interest Statement

The authors declare that the research was conducted in the absence of any commercial or financial relationships that could be construed as a potential conflict of interest.
